# Confirmatory Factor Analysis of the Oral Epithelial Dysplasia Informational Needs Questionnaire

**DOI:** 10.1111/odi.15375

**Published:** 2025-05-07

**Authors:** Waleed Alamoudi, Abdullah Alsoghier, Richeal Ni Riordain, Stefano Fedele, Stephen Porter

**Affiliations:** ^1^ Faculty of Dentistry King Abdulaziz University Jeddah Saudi Arabia; ^2^ UCL Eastman Dental Institute University College London London UK; ^3^ College of Dentistry King Saud University Riyadh Saudi Arabia; ^4^ Cork University Dental School and Hospital University College Cork Cork Ireland; ^5^ Biomedical Research Centre NIHR, University College London Hospitals London UK

**Keywords:** confirmatory factor analysis, ODIN‐Q, oral epithelial dysplasia, patient information needs, psychometric validation

## Abstract

**Objective:**

The Oral Epithelial Dysplasia Information Needs Questionnaire (ODIN‐Q) was developed to assess the informational needs of patients with oral epithelial dysplasia (OED). This study aimed to evaluate the six‐factor ODIN‐Q model to determine its psychometric properties and alignment with a theoretical framework.

**Methods:**

Confirmatory factor analysis (CFA) was conducted with 165 participants to assess the model's fit. Consensus‐based standards for selecting health measurement instruments were followed, and five participants per item in the assessment tool were required for effective CFA. Various fit indices, factor loadings and inter‐factor correlations were analysed.

**Results:**

The CFA results indicated a moderate model fit, which was consistent with other multidimensional patient‐reported instruments. The average factor loading for all 33 items was 0.58 (highest = 0.84, lowest = 0.28). Only two items with relatively low loadings (< 0.3) were related to doctors' experience and lifestyle adjustments. Additionally, the ODIN‐Q distinguished conceptually distinct domains with low inter‐factor correlations (< 0.20).

**Conclusion:**

The current six‐factor ODIN‐Q is a psychometrically sound instrument for assessing the informational needs of individuals with OED. Further cross‐cultural assessments of the ODIN‐Q are required to demonstrate its cultural sensitivity in other English‐speaking patient cohorts and globally.

## 1 Introduction

Oral epithelial dysplasia (OED) is a histological diagnosis of disturbances in cell maturation and proliferation. Although the exact mechanism of malignant transformation in OED is not well understood, it is accepted that a histological diagnosis of OED may lead to the development of oral squamous cell carcinoma (Speight 2007). Depending on the grading of the histological changes in OED, treatment may include a period of surveillance or ‘watchful waiting’ to monitor for regression or progression before considering whether surgical excision is necessary (Field et al. 2015). These periods of surveillance, investigation and therapy following the diagnosis of dysplasia have been linked to significant mental, physical and psychological burdens due to concerns about the development of cancer or its recurrence (Alsoghier et al. 2021). By providing health information, individuals can make better decisions regarding care and mitigate their worries (Gruman et al. 2010). Well‐informed patients face less uncertainty, which increases their satisfaction, strengthens their coping mechanisms and contributes to improved therapeutic results (Ormandy 2011; Neumann et al. 2011).

However, a common gap exists between the information patients need and what their physicians offer, raising the chances of ineffective shared decisions and outcomes in the patient–physician relationship (Weymann et al. 2016; Alsoghier et al. 2024). Therefore, evaluating the information needs of patients with OED is crucial and can be achieved by deploying the Oral Epithelial Dysplasia Informational Needs Questionnaire (ODIN‐Q) (Alsoghier et al. 2022). This 33‐item tool, developed in the United Kingdom, includes domains such as clinicodemographic information, disease knowledge, investigative procedures, treatments, physical and psychological aspects, healthcare systems and access to information. Lazarus and Folkman' (1984) stress, appraisal and coping theory provides a conceptual basis for developing the ODIN‐Q based on the idea that seeking information and taking proactive steps can be effective coping strategies for people dealing with challenging medical circumstances (Galloway et al. 1997; Rutten et al. 2005; White and Gallagher 2010). This framework is relevant for the diagnosis of oral precancer. The convergent validity of the ODIN‐Q was established by comparing it with a similar measure, which is consistent with the accepted guidelines for evaluating construct validity (Mokkink et al. 2019). Further evidence of construct validity was shown through hypothesis testing, revealing that patients with more pre‐existing medical conditions reported insufficient information on all items compared to those with fewer or no conditions (Mokkink et al. 2019). This aligns with the notion that individuals facing stressful situations, such as a new oral precancer diagnosis, along with other health concerns, are inclined to seek additional information as a coping method (Lazarus and Folkman 1984).

An initial assessment of the ODIN‐Q in a previous study demonstrated good content and face validity and internal consistency reliability (Alsoghier et al. 2022). However, further psychometric testing of the sufficiency of the ODIN‐Q regarding assessing information needs related to OED (i.e., structural validity) was previously recommended (De Vet 2011; Alsoghier et al. 2022). For instance, confirmatory factor analysis (CFA) offers a more advanced assessment of structural validity than other assessments of construct validity, such as hypothesis testing and cross‐cultural validity assessments (De Vet 2011). CFA shares similarities with structural equation modelling, mainly in its association with model measurements (Brown 2015). CFA provides ways to verify the fit of the proposed theoretical model for data collection, define measurement model associations and link items to their domains (Pituch and Stevens 2015). Accordingly, CFA is believed to be a very successful technique for confirming concepts in the social and behavioural sciences (Brown 2015). Using unvalidated measurement tools often leads to misleading and inaccurate results, potentially causing suboptimal planning and ineffective cessation programmes (Hewlett et al. 2007). Hence, this study aimed to conduct a CFA for the ODIN‐Q.

## 2 Materials and Methods

### 2.1 Study Design and Participants

This cross‐sectional study enrolled adult patients with OED. Based on the inclusion criteria, participants were required to be at least 18 years old, able to read and write in English, and willing to participate in the study. A summary of the study and its validation results was provided to those who agreed to participate. All participants signed an informed consent form before completing the ODIN‐Q, which included three sections: (1) socio‐demographic information, (2) level of information received and (3) preferred education methods (Table 1). The completion time for the questionnaire for laypersons was approximately 10–15 min based on its readability score (4th‐grade level).

**TABLE 1:** Content and response choices of the ODIN‐Q.

ODIN‐Q section	No of items	Components	Response choices
Socio‐demographics	7	Age, race, ethnicity, level of education, employment status and smoking and alcohol intake	Open‐ended, closed‐ended, multiple‐choice
Level of information received	33	6 categories, involving questions on knowledge about the disease, investigative procedures, treatments, physical and psychosocial aspects and medical access and information availability	0 = not applicable 1 = not at all 2 = not enough 3 = enough 4 = too much
Preferred methods of information delivery	1	Individual meetings, printed materials, audiovisual resources and group information sessions.	Multiple‐choice

Abbreviation: ODIN‐Q, oral epithelial dysplasia informational needs questionnaire.

### 2.2 Recruitment Site and Sample Size

The Royal National ENT and Eastman Dental Hospitals' Oral Medicine Unit at University College London Hospitals (UCLH) recruited eligible participants between March 2023 and December 2024. Convenient sampling was used to recruit 165 patients to complete the ODIN‐Q. The Consensus‐based Standard for the Selection of Health Measurement Instruments (COSMIN) guidelines, which state that five patients per individual item in the questionnaire are necessary for effective CFA, served as the basis for calculating the sample size (Terwee et al. 2018; Mokkink et al. 2019).

### 2.3 Ethical Considerations and Study Registration

The study procedures were carefully developed with strict adherence to the ethical principles of the Declaration of Helsinki for medical research involving human participants. The protocol was thoroughly reviewed by an independent expert, who confirmed the scientific rigor and feasibility of the study. This study was recorded by the Joint Research Office of UCLH and UCL with reference number 153912 (EDGE number) and Integrated Research Application System project ID: 318039. On 11 January 2023, the London–Surrey Borders Research Ethics Committee (reference 22/PR/1743) and the NHS Health Research Authority approved the study with favourable comments.

### 2.4 Development and Psychometric Analysis of ODIN‐Q

The stress, appraisal and coping theory introduced by Lazarus in 1984 served as the theoretical framework for developing ODIN‐Q (Lazarus and Folkman 1984). Researchers have viewed this theory as a good foundation for developing tools that meet patients' information needs, where seeking information and engaging in active behaviour are helpful coping mechanisms for a range of stressful medical conditions (Galloway et al. 1997; Rutten et al. 2005; White and Gallagher 2010). The same can also be applied to the diagnosis of oral precancer. Convergent validity was confirmed by comparing the ODIN‐Q with another instrument with a similar focus as part of the hypothesis testing process to evaluate construct validity (Mokkink et al. 2019).

### 2.5 CFA

CFA was performed to confirm the factorial structure of ODIN‐Q identified in a previous study (Alsoghier et al. 2022). Data were initially entered into Excel version 2410 and transferred to R version 4.1.1. The lavaan R package for Structural Equation Modelling, version 0.5–22 (Rosseel 2012), was used to analyse the six constructs of the ODIN‐Q level of the information received section. Model fit can be confirmed using at least three individual indices (Hair et al. 2009). No consensus has been reached on omitting items based on a specific loading level, with decisions empirically determined based on the studied construct (Knekta et al. 2019; Ondé and Alvarado 2020). However, the validity of the construct is supported by a standardised factor loading higher than 0.5 and a *p*‐value below 0.05, which reflects a strong association between items and their respective factors (McQueen et al. 2008).

## 3 Results

### 3.1 Socio‐Demographic Characteristics of Participants

Table 2 shows the socio‐demographic and clinical characteristics of the study participants (*n* = 165). The participants included 91 females (55%) and 74 males (45%) aged 25–90 years, with a mean and median age of 66 years. Based on histopathology reports, 267 dysplasia diagnoses were recorded. Dysplasia was most often mild 136 (50.93%), followed by moderate (*n* = 96; 35.95%) and severe dysplasia (*n* = 35, 13.1%). The total number of clinical sites was 194 because some participants presented with lesions at multiple sites.

**TABLE 2:** Socio‐demographic and clinical characteristics of participants (*n* = 165).

Variable	Category	Number (%)
Sex	Female	91 (55%)
	Male	74 (45%)
Age (years)	20–29	1 (0.6%)
	30–39	4 (2.42%)
	40–49	9 (5.45%)
	50–59	35 (21.21%)
	60–69	68 (41.21%)
	70–79	43 (26.06%)
	80–89	4 (2.42%)
	90–99	1 (0.6%)
Ethnicity	White (British)	88 (53.33%)
	White (other)	31 (18.78%)
	Asian or Asian British	43 (26.06%)
	Black or Black British	3 (1.81%)
Education	College or higher educational degree	98 (59.39%)
	High school diploma or less	63 (38.18%)
	Not reported	4 (2.42%)
Employment	Retired	95 (57.57%)
	Employed (full‐time)	27 (16.36%)
	Employed (part‐time)	9 (5.45%)
	Self‐employed	23 (13.93%)
	Unemployed	5 (3.03%)
	Not reported	6 (3.63%)
Smoking status	Current	22 (13.33%)
	Past	85 (51.51%)
	Never	58 (35.15%)
Alcohol consumption	Current	84 (50.9%)
	Past	26 (15.75%)
	Never	55 (33.33%)
OED histopathological examination	Mild dysplasia	136 (50.93%)
	Moderate dysplasia	96 (35.95%)
	Severe dysplasia	35 (13.1%)
OED sites	Tongue	88 (45.36%)
	Buccal mucosa	46 (23.71%)
	Gingiva	30 (15.46%)
	Hard palate	12 (6.18%)
	Floor of the mouth	10 (5.15%)
	Soft palate	5 (2.57%)
	Lips	3 (1.54%)
Associated oral disease	Oral lichen planus	115 (70.12%)
	Oral leukoplakia	28 (17.07%)
	Oral candidiasis	9 (5.48%)
	HPV‐associated lesion	7 (4.26%)
	Oral submucous fibrosis	5 (3.04%)

Abbreviation: OED, oral epithelial dysplasia.

### 3.2 CFA

CFA was performed to verify the factorial structure of the ODIN‐Q identified in a previous study (Alsoghier et al. 2022). The ODIN‐Q was divided into six theoretical constructs. Descriptive statistics, fit indices, chi‐squared tests of fit, factor loadings and interfactor correlations were computed to analyse the structural validity of the measurement tool.

#### 3.2.1 Descriptive Statistics for Factors

Table 3 summarises the central tendencies and variabilities of the scores across the six ODIN‐Q factors. Investigative tests (F2) had the highest mean score (2.70), reflecting a stronger informational need in this domain than in others. In contrast, the medical system and access to information (F6) had the lowest mean (2.11), indicating comparatively less perceived importance or relevance. Psychosocial aspects (F5) exhibited the highest variability (SD = 0.64), suggesting that respondents provided diverse responses. In contrast, the questions under general information (F1) showed the least variability (SD = 0.49), indicating more consistent responses. The scores span a broad range, with minimum values as low as 0.75 and maximum values reaching 3.33, indicating adequate dispersion across factors. These results demonstrate that the ODIN‐Q is sensitive to variability in informational needs across its domains.

**TABLE 3:** Central tendency and variability of the six factors of the ODIN‐Q.

Factor	Mean	SD	Min	Max
General information (F1)	2.45	0.49	1.10	3.30
Investigative tests (F2)	2.70	0.51	1.00	3.00
Treatments (F3)	2.42	0.50	1.00	3.17
Physical aspects (F4)	2.37	0.59	1.00	3.00
Psychosocial aspects (F5)	2.38	0.64	1.00	3.33
Medical system & access to information (F6)	2.11	0.52	0.75	3.25

Abbreviations: ODIN‐Q, oral epithelial dysplasia informational needs questionnaire; SD, standard deviation.

#### 3.2.2 CFA Fit Indices

The significant chi‐squared value (*χ* ^2^ = 947.041, df = 480, *p* < 0.001) indicated a lack of perfect alignment between the observed and model‐implied covariance matrices. Since the chi‐squared test is often sensitive to sample size, further indices were assessed (Table 4).

**TABLE 4:** Fit indices for the ODIN‐Q.

Fit measure	Value	Threshold	Interpretation
Degrees of freedom (df)	480	N/A	Sufficient degrees of freedom
Chi‐square (*χ* ^2^)	947.041	*p* > 0.05 (non‐significant)	Significant (*p* < 0.001), poor fit
Comparative fit index (CFI)	0.744	≥ 0.90	Sub‐optimal fit
Tucker–Lewis index (TLI)	0.719	≥ 0.90	Sub‐optimal fit
Root mean square error of approximation (RMSEA)	0.085	< 0.08	Moderate fit
Standardised root mean square residual (SRMR)	0.095	≤ 0.08	Sub‐optimal fit
Goodness‐of‐fit index (GFI)	0.592	≥ 0.90	Sub‐optimal fit

Abbreviation: ODIN‐Q, oral epithelial dysplasia informational needs questionnaire.

#### 3.2.3 Complete Standardised Factor Loadings

Table 5 presents the factor loadings of ODIN‐Q. Notably, the factors related to general information (F1) and psychosocial aspects (F5) were relatively consistent, whereas variability was noted for the medical system and access to information (F6). Items related to ‘*coping with disease effects*’ [Q24] and ‘chance of cure’ [Q18] strongly contributed to their respective factors, indicating well‐defined constructs. Items with weaker associations with their constructs included those related to lack of ‘*doctor experience*’ [Q26] and Q32 ‘*lifestyle adjustments*’[Q32].

**TABLE 5:** Factor loadings of the items of the ODIN‐Q.

Factor	Item	Standardised loading
General information (F1)	Q1 What is OED	0.705
	Q2 How common is it	0.625
	Q3 Risk factors	0.684
	Q4 Appearance in mouth or lips	0.571
	Q5 Is it contagious	0.574
	Q6 Role of HPV	0.421
	Q7 Disease grades and cancer risk	0.700
	Q8 Effects of smoking or drinking	0.365
	Q9 Safe level of alcohol	0.431
	Q10 Future of OED	0.537
Investigative tests (F2)	Q11 Screening and detection	0.516
	Q12 Benefits risks of tests	0.771
Treatments (F3)	Q13 If untreated	0.524
	Q14 Treatment options	0.739
	Q15 Effects on quality of life	0.757
	Q16 Self‐management	0.562
	Q17 Alternative medicine	0.400
	Q18 Chance of cure	0.790
Physical aspects (F4)	Q19 Symptom severity	0.651
	Q20 Spread to other parts	0.549
	Q21 Effects on daily activities	0.778
	Q22 Diet and nutrition	0.618
Psychosocial aspects (F5)	Q23 Fear of cancer progression	0.652
	Q24 Coping with disease effects	0.847
	Q25 Effects on social life	0.634
Medical system & access to information (F6)	Q26 Doctor experience	0.280
	Q27 Seeking second opinion	0.559
	Q28 Physical support access	0.435
	Q29 Psychological support access	0.813
	Q30 Patient support groups	0.709
	Q31 Health promotion	0.776
	Q32 Lifestyle adjustments	0.290
	Q33 Research and clinical trials	0.452

Abbreviations: HPV, human papillomavirus; ODIN‐Q, oral epithelial dysplasia informational needs questionnaire; OED, oral epithelial dysplasia.

#### 3.2.4 Inter‐Factor Correlations

Table 6 shows that most inter‐factor correlations are low (< 0.20), supporting the distinctiveness of the ODIN‐Q factors. Psychosocial aspects (F5) and physical aspects (F4) are moderately correlated (0.170), reflecting conceptual overlap.

**TABLE 6:** Inter‐factor correlations of items of the ODIN‐Q.

ODIN‐Q domains	F1	F2	F3	F4	F5	F6
General information (F1)	0.135	0.070	0.085	0.097	0.118	0.023
Investigative tests (F2)	0.070	0.073	0.067	0.077	0.080	0.017
Treatments (F3)	0.085	0.067	0.111	0.123	0.131	0.027
Physical aspects (F4)	0.097	0.077	0.123	0.201	0.170	0.039
Psychosocial aspects (F5)	0.118	0.080	0.131	0.170	0.208	0.043
Medical system & access to information (F6)	0.023	0.017	0.027	0.039	0.043	0.019

## 4 Discussion

The CFA of the ODIN‐Q conducted in this study provides valuable insights into its structural validity while confirming its clinical feasibility in capturing multiple dimensions of patient informational needs, including OED‐related general knowledge, investigative tests, treatments, physical aspects, psychosocial aspects and access to healthcare. Unlike unidimensional tools, the ODIN‐Q covers a broad spectrum of aspects, where factors are expected to be distinct rather than highly correlated (Knekta et al. 2019). Therefore, despite the suboptimal values of the fit indices, the six‐factor model remains conceptually sound, in line with its previously demonstrated strong reliability and content validity (Alsoghier et al. 2022). It also considers the limitations of statistical validation models when assessing multi‐item instruments where diverse constructs are assessed simultaneously (Byrne 2010). Additionally, it is not uncommon for health information needs' instruments to encounter similar challenges in achieving optimal CFA fit, owing to the broad range of constructs they encompass (Coulter et al. 2008).

The variability observed in these factors further underscores the sensitivity of the ODIN‐Q in capturing diverse informational needs. Psychosocial aspects exhibited the highest variability, demonstrating diverse personal coping mechanisms, social support and psychological resilience, emphasising the importance of tailoring interventions to address individual needs (Ungar and Theron 2020). In contrast, general information about OED had the least variability, indicating more consistent responses, possibly because of the universal nature of the information in this domain (Epstein and Street 2011). The broad score range for the ODIN‐Q also showed sensitivity in capturing variability to measure patients' informational requirements, aligning with recommendations for designing patient‐centred instruments that cater to diverse populations (Coulter et al. 2008).

The highest mean score was observed in the OED investigative tests, indicating that patients often prioritise information related to diagnostic processes and their implications to better understand their condition (Epstein and Street 2011). Additionally, many patients with OED and other oral precancerous changes will undergo multiple excisions as part of their care plans and may feel that their available knowledge is insufficient (Awadallah et al. 2018). In contrast, the low scores given to the medical system and access to information by many participants confirmed the often‐varying subjective perceived need for health information (Dawkins et al. 2021). Another explanation is that patients may have already received an OED diagnosis and management in a tertiary care unit, as previously addressed by primary and secondary care clinicians (Mehanna et al. 2009).

However, previous studies investigating this domain and its subcomponents have reported conflicting findings, indicating that many patients perceive access to healthcare information as an unmet or necessary need. Alsoghier et al. (2022) found that patients and clinicians identified healthcare navigation, clarity of diagnostic communication and access to specialist support as critical unmet needs. Furthermore, a psychometric evaluation of the ODIN‐Q (Alsoghier et al. 2022) demonstrated that patients frequently reported gaps in access to information about clinical trials, patient support groups and secondary opinions, reinforcing the importance of this domain despite its low scores in this study. These findings highlight the potential variability in patient preferences, suggesting that, although some may feel that their informational needs have been met through previous healthcare interactions, others experience ongoing gaps in understanding and accessing medical resources, warranting further exploration.

In psychometric evaluations, instruments often exhibit suboptimal fit index validations. Researchers frequently justify these findings by emphasising the instrument's theoretical foundation, practical utility and complexity of the measured constructs. For instance, researchers have encountered challenges in achieving ideal fit indices in developing health‐related quality of life measures such as emPHasis‐10 for patients with pulmonary hypertension. Despite these challenges, the instrument was deemed valuable because of its comprehensive coverage of the construct and applicability in diverse settings (Yorke et al. 2014). Similarly, researchers validating the Chronic Heart Failure Health‐Related Quality of Life Questionnaire reported certain suboptimal fit indices. However, they justified the retention of specific items based on their clinical significance and the overall content validity of the instrument, ensuring its relevance to the target population (Zhao et al. 2024). These examples underscore the importance of balancing statistical rigour with theoretical and practical considerations.

Notably, the reported RMSEA (0.085) exceeded the adopted threshold of 0.08 and remains appropriate for multidimensional scales (Browne and Cudeck 1992). This value is consistent with other multidomain patient‐reported measures, where slight deviations from the ideal model fit are often attributed to the diversity of patient needs rather than measurement flaws (Kline 2023). Additionally, the SRMR (0.095), whereas above the threshold of 0.08, does not necessarily indicate a significant measurement problem but rather the need for minor revisions in item wording and factor structure.

The inter‐factor correlations provided further support for maintaining the six‐factor structure of the ODIN‐Q. Most correlations remained below 0.20, indicating that the factors were conceptually distinct, which was expected given the diverse nature of patient informational needs (Della et al. 2013). Although the medical system and access factors had weaker inter‐factor correlations, this does not necessarily imply poor construct validity. Instead, it reflects the unique nature of access‐related concerns that may not always be strongly correlated with knowledge‐ or symptom‐related factors (Ng 2013). Other studies on patient information needs have also found that system‐related constructs often behave differently in statistical models owing to external influences such as healthcare accessibility, literacy levels and individual patient experiences (Zikmund‐Fisher et al. 2010). Therefore, the lower correlations observed in the ODIN‐Q medical system and access domain did not reduce its clinical relevance. Instead, they underscore the complexity of assessing patients' experiences with healthcare systems. Retaining these items, even with moderate statistical performance, ensures that the ODIN‐Q captures a comprehensive picture of patients' informational needs, particularly for individuals facing barriers to healthcare access and navigation (Scott et al. 2002).

This study provides evidence of the conceptual overlap between some factors. Notably, information concerning psychosocial and physical aspects was moderately correlated (0.170), suggesting that physical health concerns influence psychosocial well‐being, as observed in other studies on health‐related quality of life (Epstein and Street 2011). This relationship aligns with the understanding that physical and psychological domains are often interconnected in health contexts, particularly in individuals managing chronic or potentially malignant conditions (Chapman et al. 2005). Similarly, psychosocial aspects demonstrate slightly stronger correlations with other factors, reflecting the central role of psychosocial considerations in patients' experiences and information needs (Pourhaji et al. 2023).

The ODIN‐Q is a rigorously developed instrument that has undergone extensive reliability and validity testing, making it a valuable tool for assessing the diverse information needs of patients with OED. The broad response range and variability in the factor scores demonstrate its sensitivity in measuring the perceived importance of different informational needs. Future research should focus on further validation in diverse populations to ensure that the ODIN‐Q is applicable across different clinical settings and patient cohorts in the United Kingdom. Additionally, a longitudinal approach to assessing the informational needs of patients with OED is essential for understanding how patient concerns evolve throughout the care pathway from diagnosis to long‐term management. Studies using patient‐reported outcome measures and patient‐reported experience measures have demonstrated the importance of capturing evolving patient concerns, with findings showing that information needs related to diagnosis and prognosis often give way to treatment and survivorship concerns (Di Maio et al. 2022). This is particularly relevant for OED, where patients frequently undergo multiple excisions and long‐term surveillance, making tailored, stage‐specific information critical for patient engagement and adherence to follow‐up care (Mehanna et al. 2009).

This study had some limitations. First, the sample was recruited from a single dental hospital; thus, the findings may not be generalisable to a broader population. Differences in health literacy, cultural background and access to healthcare can influence responses and affect a tool's applicability across various contexts. Second, this cross‐sectional study only provides a snapshot of informational needs at a single time point. It does not capture the changes in patients' informational needs or experiences over time, thus limiting its ability to assess the tool's longitudinal utility. Third, the participants may have provided socially desirable responses, particularly in domains related to knowledge and behaviour. Such biases could affect the accuracy of the results and mask the true informational gaps. Fourth, although the study focused on confirming the factor structure of the ODIN‐Q, additional aspects of validation, such as predictive validity, criterion‐related validity and test–retest reliability, were not addressed, leaving specific psychometric properties unexplored.

## 5 Conclusions

This study provided a comprehensive psychometric evaluation of the ODIN‐Q, confirming its clinical utility and validity for assessing the diverse informational needs of patients with OED. The ODIN‐Q effectively distinguishes distinct informational domains, including general knowledge, investigative tests, treatments, physical aspects, psychosocial aspects, medical systems and access to information, making it a valuable tool for patient‐centred care. Although the statistical fit indices suggest minor areas of improvement, such as refining or subdividing some items within the medical system and access to the information domain, the six‐factor model remains conceptually sound, reflecting the multidimensional nature of patient needs. Future studies should continue to validate this tool across diverse populations and explore its longitudinal applicability in assessing evolving patient information needs.

## Author Contributions

**Waleed Alamoudi:** conceptualization, investigation, funding acquisition, writing – original draft, methodology, validation, writing – review and editing, software, formal analysis, project administration, data curation. **Abdullah Alsoghier:** conceptualization, writing – review and editing. **Richeal Ni Riordain:** conceptualization, investigation, methodology, validation, writing – review and editing, supervision. **Stefano Fedele:** conceptualization, investigation, writing – review and editing, supervision, resources. **Stephen Porter:** conceptualization, investigation, funding acquisition, methodology, writing – review and editing, project administration, supervision, resources.

## Conflicts of Interest

The authors declare no conflicts of interest.

## Data Availability

The data that support the findings of this study are available on request from the corresponding author. The data are not publicly available due to privacy or ethical restrictions.
